# Long-segment common carotid occlusion presenting with limb-shaking transient ischemic attack: Case report

**DOI:** 10.3389/fsurg.2022.1028004

**Published:** 2023-02-15

**Authors:** Qingjun Jiang, Jun Bai, Shaojie Nie, Jie Jin, Lefeng Qu

**Affiliations:** Department of Vascular and Endovascular Surgery, Changzheng Hospital, Second Military Medical University, Shanghai, China

**Keywords:** limb-shaking TIA, common carotid artery occlusion, carotid endarterectomy, visual impairment, corpus striatum

## Abstract

**Background:**

Limb-shaking transient ischemic attack (LS-TIA) is a rare manifestation of carotid artery occlusion. Common carotid artery occlusion (CCAO) is a relatively rare condition, and both its natural history and recommendations for treatment are still unclear.

**Case description:**

A 67-year-old female suffered from transient episodes of unilateral limb shaking. Computer tomographic angiography (CTA) showed long-segment occlusion of the right common carotid artery. Computer tomographic perfusion (CTP) demonstrated hypoperfusion of the corpus striatum, which suggests that hemodynamic failure is a potential mechanism underlying the LS-TIA secondary to common carotid artery occlusion. The occlusion was successfully recanalized by retrograde common carotid endarterectomy, and the episodes of left limb shaking disappeared after surgery.

**Conclusions:**

The occlusion was successfully recanalized by retrograde common carotid endarterectomy, and the episodes of left limb shaking disappeared after surgery. Hypoperfusion of the corpus striatum might be a potential mechanism underlying the LS-TIA secondary to common carotid occlusion.

## Introduction

Limb-shaking transient ischemic attack (LS-TIA) is a rare manifestation of severe stenosis or occlusion of the internal carotid artery (ICA) (1). It is characterized by transient involuntary, repetitive jerking or trembling movements of the limbs. Although the compromised hemodynamics secondary to an occlusive disease has been suggested to be the cause of LS-TIA, the exact pathophysiology underlying the limb-shaking is not clear. LS-TIA seriously affects patients' quality of life and increases the risk of future stroke (2). Therefore, it is very important to treat limb shaking as early as possible.

Common carotid artery occlusion (CCAO) is a relatively rare condition, and both its natural history (3) and recommendations for treatment are still unclear (4). LS-TIA caused by CCAO has never been reported. We report a patient with LS-TIA caused by hypoperfusion of the corpus striatum, which is secondary to the long-segment CCAO; this patient was successfully treated by ring-stripping retrograde common carotid endarterectomy (RS-CEA).

## Case description

**Figure 1:**
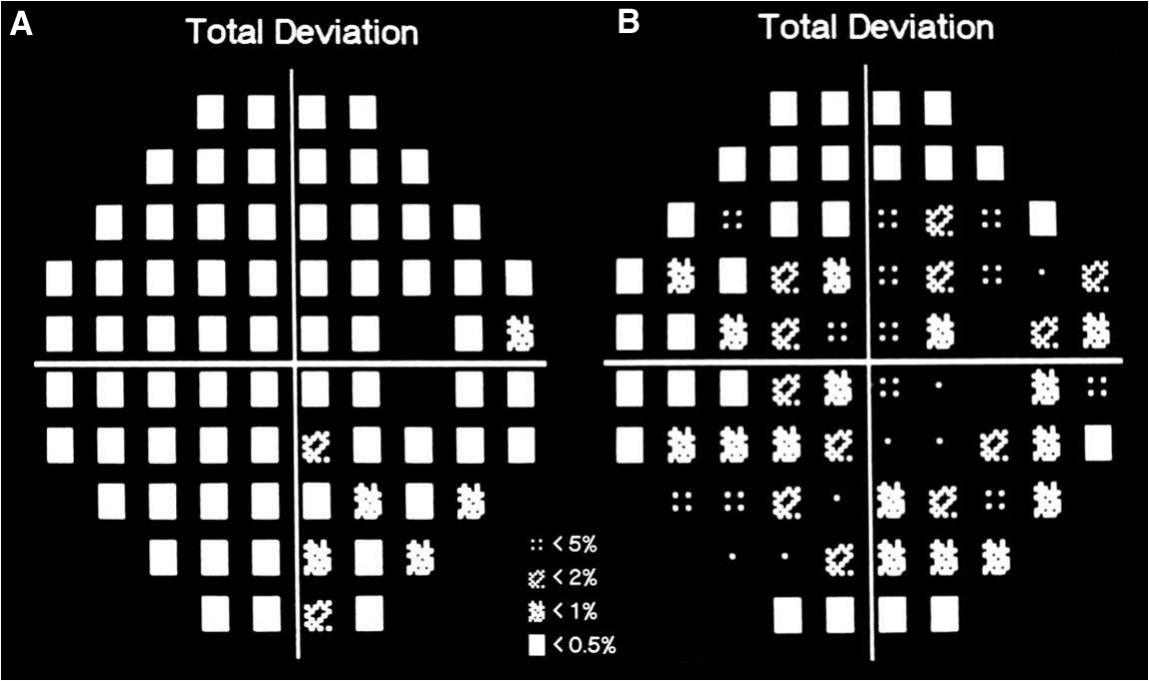
The total deviation of right eye. The visual field test showed better result of the right side than that was pre-operation. Black squares indicate deficit of the visual fields. (**A**) Pre-operation. (**B**) Post-operation.

A 67-year-old female suffered from transient episodes of unilateral limb shaking for two years. Two years ago, she initially developed shaking of her left hand, arm and leg. Meanwhile, she had blurred vision, but her symptoms were untreated. Over the past month, the left limb shaking was aggravated, and occurred in combination with jerky and arrhythmic shaking movements of the neck that spread to the left upper and lower limbs. These episodes occurred 3–4 times a day and lasted for 2–3 min each. These attacks occurred when the patient arose from a sitting position. When experiencing these limb-shaking episodes, the patient felt that her left limbs were weak and was unable to stand steadily. These symptoms were resolved within seconds of her lying down. She had a history of intermittent claudication involving the left lower limb, hypertension and diabetes and was taking anti-hypertensive medication, oral hypoglycemic agents, platelet inhibition medications and statins. On admission, her blood pressure was 170/130 mm Hg. Physical examination did not indicate any neurological deficits. The International Standard (IS) visual acuity chart was used to test the patient's visual acuity, which was 0.5 on the right side and 0.6 on the left (the best score was 1.5, higher values = better function). Automated static perimetry (Octopus 300, program G1, Haag-Streit, United States) was used to test the visual field. The mean deviation (MD) was −16.05 dB in her right eye and −9.23 dB in her left eye (higher MD values indicate greater deficits of the visual fields). The total deviation in the right eye is shown in Figure 1A.

**Figure 2:**
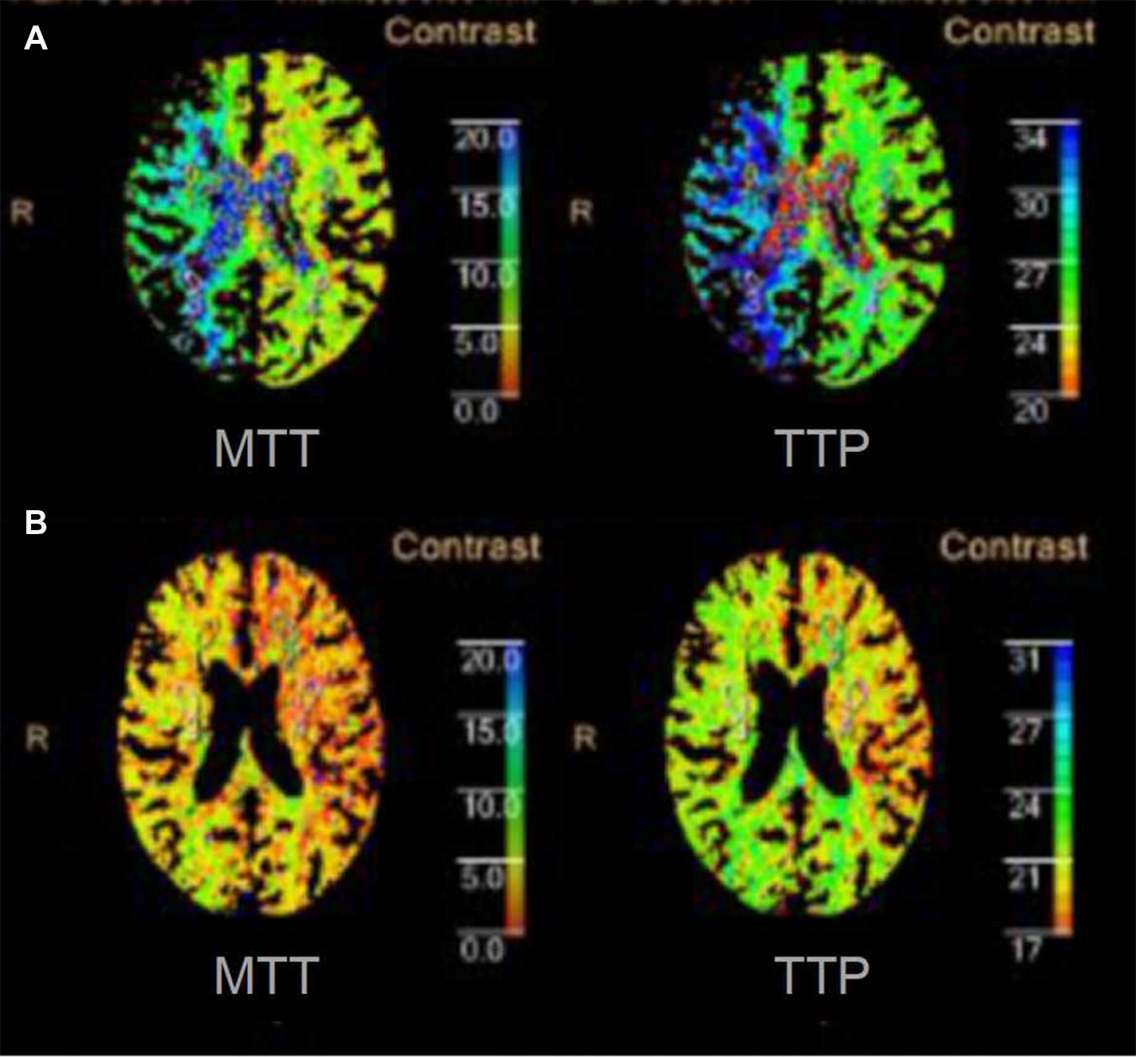
Computed tomography perfusion of corpus callosum plane. (**A**) Preoperative CTP showed that the perfusion of right cerebral hemisphere was lower than the left. (**B**) Postoperative CTP showed the perfusion of right cerebral hemisphere was better than that was pre-operation.

**Figure 3:**
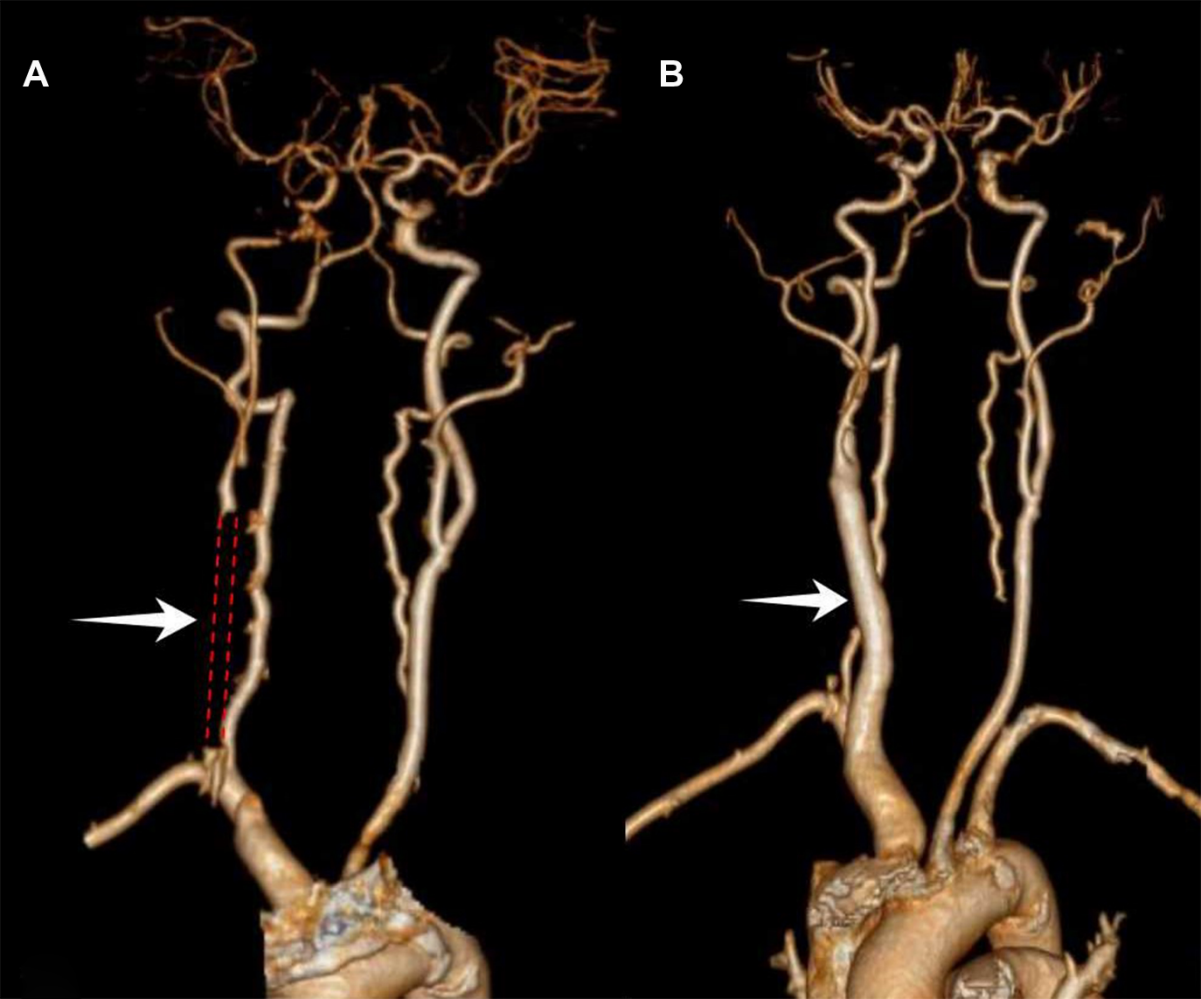
Computer tomographic angiography of carotid artery. (**A**) Preoperative CTA indicated the right CCA long-segmental occlusion and the right ICA initial-segmental occlusion. (**B**) The one-year follow-up CTA indicated persistent patency of the CCA and ICA.

Magnetic resonance imaging (MRI) showed that there was no evidence of acute infraction. Computer tomographic perfusion (CTP) showed that the perfusion of the right cerebral hemisphere was lower than that of the left (Figure 2A). The values of mean transit time (MTT) and time to peak (TTP) were maximal in the corpus striatum region; the values were 17.7 and 35.3, respectively (lower values indicate better perfusion). Magnetic resonance angiography (MRA) showed that the patient had a complete circle of Willis. Doppler ultrasound indicated right ICA initial segment occlusion. Computer tomographic angiography (CTA) revealed occlusion of the right CCA long segment and the right ICA initial segment (Figure 3A). The right CCAO was thought to be a significant contributor to the patient's limb-shaking TIA symptoms. The diagnosis of low flow TIA was made. Because medical therapy (platelet inhibition medications and statins) could not relieve her symptoms, surgical revascularization of the right CCA was performed in this patient.

**Figure 4:**
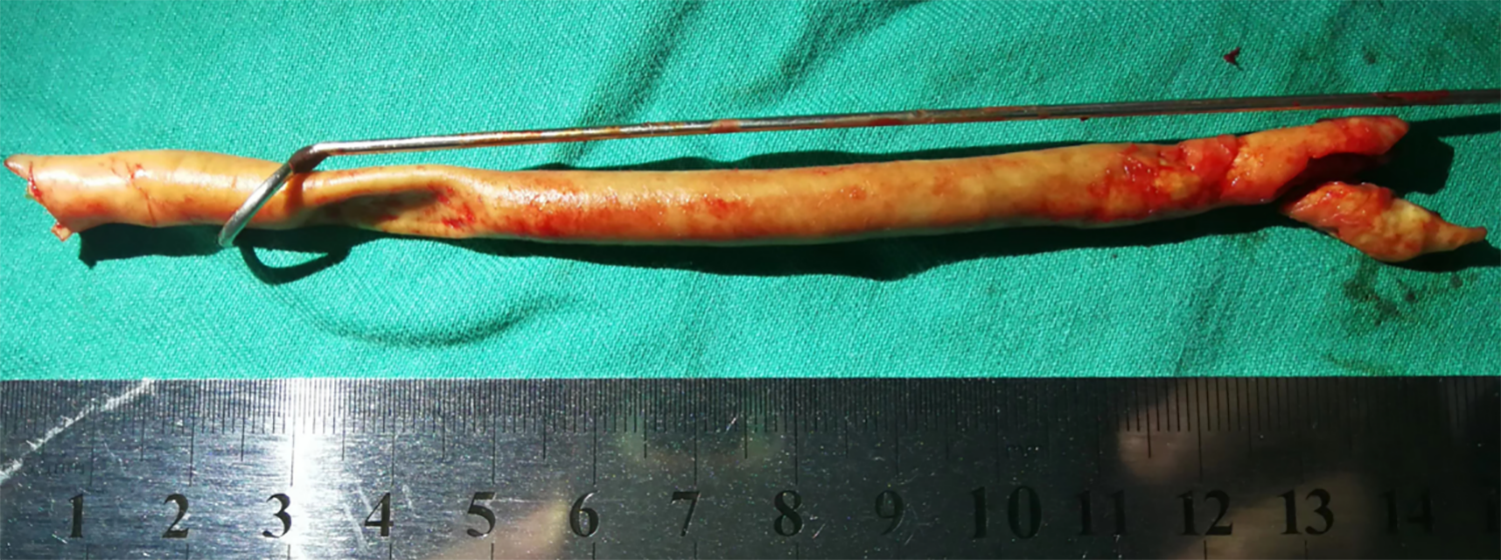
The CCA plaques were completely removed, and the length was 13 cm.

The patient underwent RS-CEA, which was performed in a hybrid operating room under general anesthesia. The ICA plaque was removed by routine eversion endarterectomy. The CCA plaque was removed by ring-stripper endarterectomy. Under the guidance of a fluoroscopic roadmap and bony landmarks, the stripper reached and exceeded the proximal occluded region of the CCA. Then we used the ring-stripper to slowly perform endarterectomy in a retrograde fashion. The CCA plaques were completely removed, and the length was 13 cm (Figure 4). There was no residual stenosis, intimal discontinuity, or intracranial vascular embolism.

The patient made a full recovery. The episodes of left limb shaking disappeared after the procedure. She felt that her visual field was brighter than before. On the sixth postoperative day, the patient underwent visual acuity and field testing. The visual acuity test showed better results on the right side (right 0.8, left 0.6) than pre-operation. The visual field test showed improvement in the results on the right side (right MD −9.13 and left MD −9.32) compared to the results of the pre-operative evaluation. The total deviation of the right eye is shown in Figure 1B. On the seventh postoperative day, CTP showed that the perfusion of the right cerebral hemisphere was increased. The values of MTT and TTP of the caput nuclei caudati were 4.3 and 20.4, respectively (Figure 2B). The patient was treated with 100 mg aspirin and 10 mg rosuvastatin qd after discharge. The four-year follow-up CTA demonstrated persistent patency of the CCA and ICA (Figure 3B). To date, the patient has not experienced recurrent involuntary movements since the operation.

## Discussion

Treatment of LS-TIA includes surgical revascularization and medical modalities, but most of the patients are still treated conservatively with medication (5). To date, aggressive medical treatment with antiplatelet agents, statins, and risk-factor modification remains the first choice for patients with LS-TIA. These patients may benefit from revascularization therapy beyond aggressive medical management alone. Surgical management can be considered if the patient remains refractory to medical therapy. In most cases, LS-TIA is predominantly caused by stenosis or occlusion of the ICA. Carotid endarterectomy is a good choice to relieve symptoms and reduce the risk of stroke. In our presented case, the key to the successful resolution of this case was to remove the CCA plaque in the long segment.

The incidence of CCAO is approximately 3% in patients who undergo angiography for symptomatic cerebrovascular disease (6). To date, guidelines have not recommended an optimal surgical procedure for CCAO (4), which remains controversial. Endovascular therapy and open surgery have been reported as means of treating CCAO, such as carotid stenting, carotid-carotid artery crossover bypass and extracranial-intracranial (EC-IC) bypass surgery. However, when performing CCA stenting, it is usually challenging to pass the guidewire through chronic long-segmental occluded lesions, and the risk of vascular dissection and cerebral infarction is high. Carotid-carotid artery crossover bypass is an option for the treatment of CCAO, but the extra-anatomical approach and infections of the synthetic vascular grafts are the problems association with this surgical procedure. The Carotid Occlusion Surgery Study (COSS) compared medical therapy to EC-IC bypass in patients with recently symptomatic carotid occlusion (7). However, thirty-day rates for ipsilateral ischemic stroke in the surgical group were significantly higher than in the nonsurgical group (14.4% vs. 2.0%).

After a full pre-operative assessment, we performed RS-CEA for this patient instead of carotid-carotid artery crossover bypass or EC-IC bypass. The advantage of RS-CEA is that it can simultaneously treat long-segmental CCAO and initial-segmental occlusion of ICA with a relatively small neck incision. A retrospective study showed that RS-CEA was an effective treatment for chronic CCAO (8). Although RS-CEA is effective in the treatment of CCAO, it may cause vascular rupture and restenosis. This technique requires a great deal of surgical training and sufficient endovascular skill. Our center has performed RS-CEA on selected cases of long-segmental CCAO since 2008.

Although compromised hemodynamics secondary to an occlusive disease has been suggested as the cause of LS-TIA, the exact pathophysiology underlying limb-shaking is not clear. The pathophysiology of LS-TIA can be explained by the hypoperfusion theory. Tatemichi et al. (9) found significant hypoperfusion of the right dorsofrontal and upper rolandic regions contralateral to the shaking limb in a 63-year-old patient. In this case, we found hypoperfusion of the right corpus striatum. These reversible deficits in cerebral blood flow, which were maximal in the corpus striatum region, are consistent with low perfusion in the border zone territory. The physiological function of the striatum is to maintain the stability of movement. Involuntary movement, such as hand-foot tremor, can be caused by striatal dysfunction. This suggests that hemodynamic failure is the potential mechanism underlying LS-TIA secondary to CCAO. It is worth noting that the patient's visual acuity and visual field increased after surgery. This may be explained by the improvement in the blood flow in the ophthalmic artery (10).

## Conclusions

In conclusion, our case suggests that hemodynamic failure may be the potential mechanism underlying LS-TIA secondary to CCAO. The RS-CEA is an effective treatment for long-segment CCAO.

## Data Availability

The original contributions presented in the study are included in the article/Supplementary Material, further inquiries can be directed to the corresponding author/s.
